# Dynamic contrast-enhanced perfusion parameters in ovarian cancer: Good accuracy in identifying high HIF-1α expression

**DOI:** 10.1371/journal.pone.0221340

**Published:** 2019-08-22

**Authors:** Auni Lindgren, Maarit Anttila, Suvi Rautiainen, Otso Arponen, Kirsi Hämäläinen, Mervi Könönen, Ritva Vanninen, Hanna Sallinen

**Affiliations:** 1 University of Eastern Finland, Faculty of Health Sciences, School of Medicine, Institute of Clinical Medicine, Obstetrics and Gynecology, Kuopio, Finland; 2 Department of Obstetrics and Gynecology, Kuopio University Hospital, Kuopio, Finland; 3 Cancer Center of Eastern Finland, University of Eastern Finland, Kuopio, Finland; 4 Department of Clinical Radiology, Kuopio University Hospital, Kuopio, Finland; 5 Department of Clinical Pathology and Forensic medicine, Kuopio University Hospital, Kuopio, Finland; 6 University of Eastern Finland, Faculty of Health Sciences, School of Medicine, Institute of Clinical Medicine, Pathology and Forensic medicine, Kuopio, Finland; 7 Department of Clinical Neurophysiology, Kuopio University Hospital, Kuopio, Finland; 8 University of Eastern Finland, Faculty of Health Sciences, School of Medicine, Institute of Clinical Medicine, Clinical Radiology, Kuopio, Finland; Medical University of Vienna, AUSTRIA

## Abstract

**Background:**

Hypoxia significantly influences treatment response and clinical outcome in solid tumors. A noninvasive marker for hypoxia will help physicians in treatment planning and encourage the efficient use of hypoxia targeted therapies. The purpose of this study was to investigate whether pharmacokinetic dynamic contrast-enhanced (DCE) perfusion parameters are associated with a specific marker of hypoxia, hypoxia-inducible factor 1 alpha (HIF-1α) in ovarian cancer (OC).

**Materials and methods:**

Thirty-eight patients with primary OC were enrolled in this prospective study approved by the local ethical committee. Patients underwent dynamic gadolinium-enhanced 3.0 T MRI as part of their staging investigations. Pharmacokinetic perfusion parameters, including a rate constant for transfer of contrast agent from plasma to extravascular extracellular space (EES) (K^trans^) and a rate constant from EES to plasma (K_ep_), were measured by drawing two types of regions of interest (ROIs): a large solid lesion (L-ROI) and a solid, most enhancing small area (S-ROI) (NordicICE platform). Tissue samples for immunohistochemical analysis were collected during surgery. Kruskal–Wallis, Mann–Whitney U and Chi-square tests were used in statistical analyses. Receiver Operating Characteristic curve analyzes were done for DCE parameters to discriminate high HIF-1α expression.

**Results:**

Pharmacokinetic perfusion parameters K^trans^ and K_ep_ were inversely associated with HIF-1α expression (K^trans^ L-ROI *P* = 0.021; K^trans^ S-ROI *P* = 0.018 and K_ep_ L-ROI *P* = 0.032; K_ep_ S-ROI *P* = 0.033). K^trans^ and K_ep_ showed good accuracy in identifying high HIF-1α expression (AUC = 0.832 K^trans^ L-ROI; 0.840 K^trans^ S-ROI; 0.808 K_ep_ L-ROI and 0.808 K_ep_ L-ROI).

**Conclusion:**

This preliminary study demonstrated that pharmacokinetic DCE-MRI perfusion parameters are associated with the hypoxia specific marker, HIF-1α in OC. DCE-MRI may be a useful supplementary tool in the characterization of OC tumors in a staging investigation.

## Introduction

Ovarian cancer (OC) is one of the most lethal malignancies in women. Although the mortality rate has declined by 33% between 1976 and 2015 with reductions in both incidence and mortality, nonetheless the five-year survival rate is as low as 47% [1]. Extensive research efforts are being expended to improve the patient’s survival. Tumor hypoxia is a novel, potentially useful, target for anti-cancer drugs [2–6]. It is now well established that hypoxia is an important component of the tumor microenvironment. The growing tumor requires more oxygen and when it reaches a size of 2mm^3^, hypoxia occurs. Hypoxia-inducible factor 1 (HIF-1) is a heterodimer transcription factor formed from HIF-1α and HIF-1β subunits. HIF-1β is a stable subunit, being constitutively expressed while HIF-1α is increasingly expressed in hypoxic situations [7] and thus it has been proposed as a molecular marker of hypoxia [8]. HIF-1α functions as a key regulator of the cellular response to hypoxia, modulating the expression of genes involved in processes such as metabolism, proliferation, angiogenesis and apoptosis [7,9,10]. HIF-1α has shown promise as a prognostic factor in OC [11,12]. Hypoxia plays an important role in chemotherapy and radiotherapy resistance. Although OC is sensitive to chemotherapy, 70% of patients relapse [13]. This has led to studies investigating which kind of combinations of drugs or other treatment modalities would benefit patients more. HIF-1 inhibition seems to be a promising target. There are different mechanisms of action that lead to decreased HIF-1 transcriptional activity: decreased HIF-1α synthesis, decreased HIF-1 DNA binding, increased HIF-1α degradation and decreased HIF-1α transactivation [14–18]. There are different classes of drugs effecting for these mechanisms. Several drugs have been promising in preclinical studies and are already in early clinical trials [4–6]. Current HIF-1 inhibitors suffer a nonspecific mode of action and because HIF-1 regulation comprises very complex cascade it has been very challenging task to design selective HIF-1 inhibitor, but this will be the goal in the future. It would be important to find a marker that would be predictive of hypoxia and thus help to guide treatment e.g. in devising individualized biological treatments.

Conventional magnetic resonance imaging (MRI) is applied in the preoperative imaging of indeterminate ovarian tumors due to its superior soft tissue extraction. Dynamic contrast enhanced (DCE) sequence, utilizing contrast agent extravasation from blood flow to tissues, may not only be used to characterize benign lesions from malignant tumors [19,20] but may also provide additional information on the tumor prognosis [21,22] and treatment response [23–25]. Pharmacokinetic perfusion parameters reflect the circulation physiology in the microvasculature and give quantitative parameters to compare flow and vessels permeability properties [26–28].

Few studies have correlated DCE parameters to measures of tumor oxygenation levels such as HIF-1α. In gliomas, there seems to be a positive correlation between HIF-1α and DCE perfusion parameters [29–31], and other studies an inverse correlation has been reported [25,32]. We hypothesized that DCE-MRI could provide a noninvasive tool for the identification of tumor hypoxia also in ovarian cancer. The aim of this study was to investigate whether DCE pharmacokinetic perfusion parameters in OC would be associated with a specific marker of hypoxia, HIF-1α.

## Materials and methods

### Study protocol and patients

This prospective single-institution study was conducted between 2011 and 2014. The Northern Savo research ethical committee approved the study protocol (approval number 5302473) and written informed consent was obtained from all patients. A total of 38 patients was enrolled. Power calculations were not performed prospectively because no prior knowledge of the DCE parameters and HIF in OC existed, and in other cancers preliminary studies have been conducted with similar size [25,29,31,33]. The inclusion criteria included a clinical diagnosis of primary OC, primary fallopian tube cancer or primary peritoneal carcinoma, as they are diagnosed and treated as one entity and measurable disease at staging computed tomography (CT). The exclusion criteria were contraindications to MRI or to gadolinium contrast agents. All patients underwent diagnostic 3.0 T MRI before any treatment. Samples from tumors to be subjected to immunohistochemical analysis were collected during surgery. Cancers were staged using the International Federation of Gynecology and Obstetrics (FIGO) guidelines. The histological type and grade of the tumors were evaluated according to World Health Organization criteria. An experienced multidisciplinary team chose the modality of first-line treatment (surgery, n = 34; neoadjuvant chemotherapy before surgery, n = 5). Adjuvant chemotherapy after the operation was paclitaxel–carboplatin (n = 37), one patient received carboplatin monotherapy for her stage IA disease. Detailed characteristics of the patients are presented in Table 1.

**Table 1:** Patients characteristics (n = 30) and distribution according to either low or high hypoxia-inducible factor 1 alpha (HIF-1α) expression.

Variable	HIF-1α low	HIF-1α high	p
**Age** *	67 [49–86]	57 [47–72]	0.206
**BMI** *	26 [17–34]	27 [24–40]	0.188
**CA 12–5** *	369 [16–1673]	483 [69–952]	0.957
**Disease variable**	n (%)	n (%)	
**Grade**			0.892
1	1 (3.3)	0 (0.0)	
2	8 (26.7)	2 (6.7)	
3	16 (53.3)	3 (10.0)	
**FIGO stage**			0.900
1	4 (13.3)	1 (3.3)	
2	2 (6.7)	0 (0.0)	
3	11 (36.7)	2 (6.7)	
4	8 (26.7)	2 (6.7)	
**Histology**			0.952
High grade serous	17 (56.7)	3 (10.0)	
Endometrioid	4 (13.3)	1 (3.3)	
Mucinous	0 (0.0)	0 (0)	
Clear cell	1 (3.3)	0 (0)	
Other histology	3 (10.0)	1 (3.3)	
**Ascites**			0.968
No	5 (16.7)	1 (3.3)	
Yes	20 (66.7)	4 (13.3)	
**Residual tumor at primary surgery**			0.348
No	14 (46.7)	1 (3.3)	
</ = 1cm	9 (30.0)	3 (10.0)	
>1cm	2 (6.7)	1 (3.3)	
**Tumor recurrence**			0.692
No	10 (40.0)	1 (4.0)	
Yes	12 (48.0)	2 (8.0)	
**Platinum sensitivity**			0.428
Sensitive	20 (66.6)	3 (10.0)	
Resistance	5 (16.7)	2 (6.7)	
**Chemotherapy response**			0.395
Complete response	20 (66.6)	3 (10.0)	
Partial response	2 (6.7)	0 (0)	
Stable disease	0 (0)	0 (0)	
Progressive disease	3 (10.0)	2 (6.7)	

HIF-1α expression has been dichotomized into low and high using the median immunoreactive score as the cutoff value

*Results are median with range in square brackets

BMI = body mass index, FIGO = the International Federation of Gynecology and Obstetrics

### Imaging protocol

MRI was performed with a 3.0 T scanner (Philips Achieva 3.0T TX, Philips N.V., Eindhoven, The Netherlands) with a body coil (Sense-XL-Torso) covering the whole abdomen from the lower thorax to the symphysis. The structured MRI protocol included transaxial, sagittal, and coronal T2-weighted sequence (repetition time (TR) 651 ms, echo time (TE) 80 ms, flip angle 90°, resolution 0.7 mm x 0.7 mm x 0.5 mm), transaxial fat-suppressed spectral attenuated inversion recovery *(*SPAIR) sequence (TR 744 ms, TE 70 ms, flip angle 90°), DUAL- fast field echo (FFE) sequence (TR 180 ms, TE 1.15 ms outphase and 2.30 ms inphase, flip angle 55°, resolution 1.3 mm x 1.3 mm x 5.0 mm), diffusion weighted image (DWI) sequence (TR 490 ms, TE 48 ms, flip angle 90°, resolution 1.8 mm x 1.8 mm x 5.0 mm), DCE sequences GD dyn eThrive SENSE (TR 3.8 ms, TE1.8 ms, flip angle 10°, resolution 0.9 mm x 0.9 mm x 5.0 mm, at 6.7s intervals a total of 23 timeframes) and T1w post-contrast images (TR 6.9ms, TE 3.5ms, flip angle 10°, resolution 1.5 mm x 1.5 mm x 3.0 mm). During DCE image acquisition, the contrast agent gadoterate meglumine (Dotarem 279.3 mg/ml, Guerbet, France) was injected intravenously as a bolus dose of 0.1 mmol/kg at a rate of 4 ml/s using an MRI-compatible power injector (Optistar Elite, Covidien, Los Angeles, CA, USA), followed by a 20 ml flush of 0.9% sodium chloride solution.

### Image analysis

**Fig 1:**
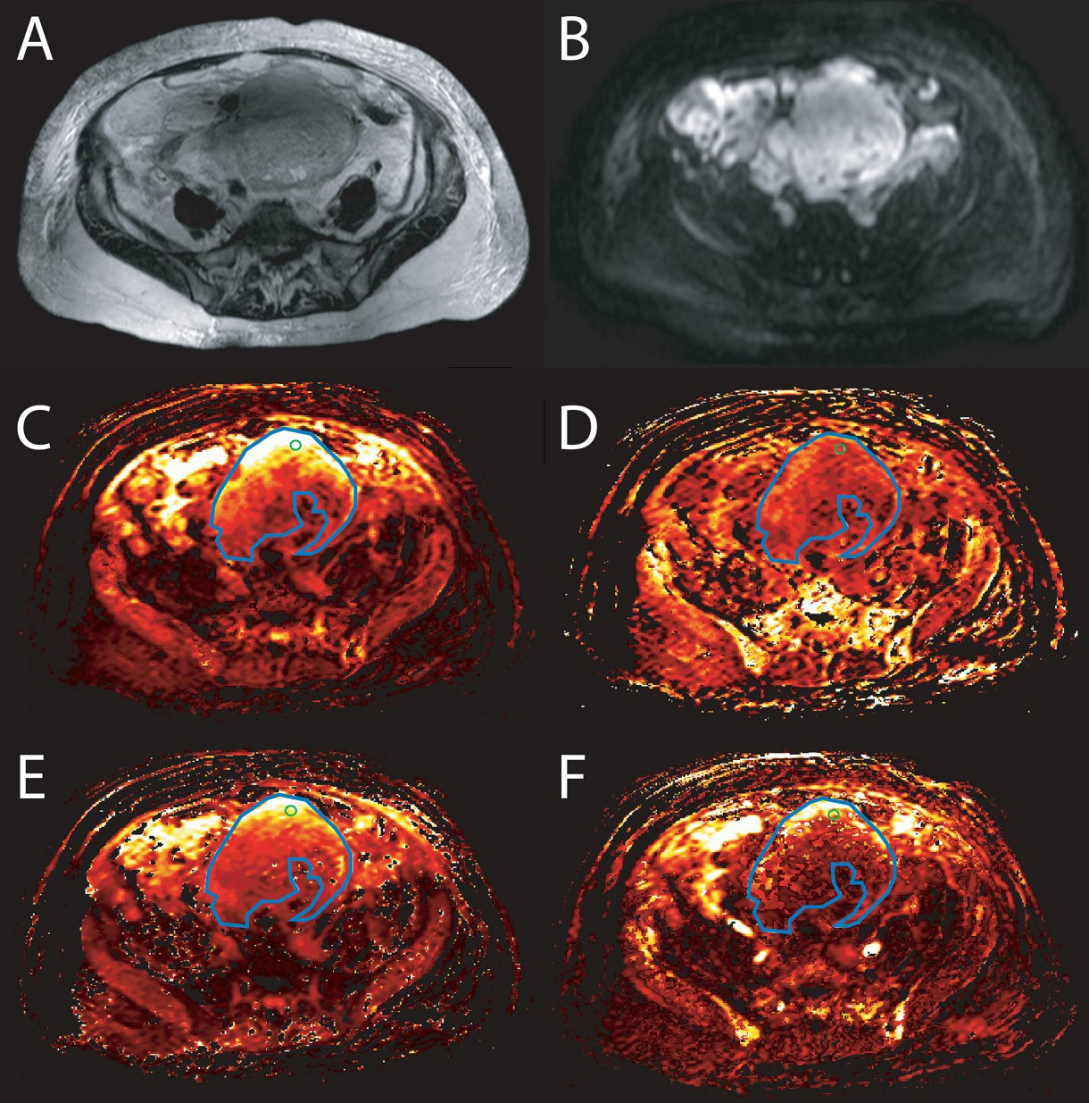
Image of a woman with a high grade ovarian carcinoma, FIGO stage IIIC. A) A T2-weighted sequence illustrating a large cystic-solid tumor, B) in diffusion weighted image (DWI) tumor is bright. There is large region of interest (ROI) marked in blue color and a small ROI in green color drawn on the big solid part of the tumor (cystic and small solid parts between cysts was avoided). The ROIs were replicated onto color encoded C) K^trans^ (a rate constant for transfer of contrast agent from plasma to the extravascular extracellular space (EES)) map, D) K_ep_ (a rate constant from EES to plasma) map, E) V_e_ (contrast agent distribution volume) map and F) V_p_ (plasma volume fraction) map.

Two observers (AL and OA), with 4 and 3 years of experience in gynecological imaging, evaluated all MR- and DCE- sequences blinded to the histopathological information using a Sectra PACS workstation (IDS7, Version 15.1.20.2, Sectra AB, Linköping, Sweden) and special imaging software NordicICE (version: 2.3.13, NordicNeuroLab, Bergen, Norway). In unclear cases a senior radiologist (SR) with 12 years of experience in gynecological imaging was available for consultation. Image analysis has been described in detail previously [22]. The perfusion parameter maps were generated automatically with NordicICE using pharmacokinetic modeling of contrast kinetics according to the Tofts model [28]. Motion correction was done automatically at first. The perfusion was quantified by determining the arterial input function (AIF) by drawing a small AIF ROI onto the common or external iliac arteries. According to clinical practice at the time of study designing, B1 maps and T1 mapping were not used. Four quantitative parameters were measured as follows; 1. K^trans^, a rate constant for the transfer of contrast agent from plasma to the extravascular extracellular space (EES), 2. K_ep_, a rate constant from EES to plasma; 3. V_e_, contrast agent distribution volume and 4. V_p_, fractional plasma volume. All measurements were obtained using the transaxial images showing the largest solid tumor diameters in the ovary. Two regions of interests (ROIs) with different sizes were used; first a large ROI (L-ROI) was drawn free-hand to cover the whole solid tumor area, excluding cystic, necrotic and vascular areas. A small circle ROI (S-ROI), with a set size of 15×15 pixels, was then placed on the area considered to be the most solid and intensively enhancing. T2w-, T1w-, DWI-, and contrast-enhanced T1w- images were all available for tumor localization and ROI delineation. The ROIs were then replicated to the DCE parameter maps illustrated in Fig 1. Mean values were registered for each DCE parameter in the analyses.

### Immunohistochemistry

**Fig 2:**
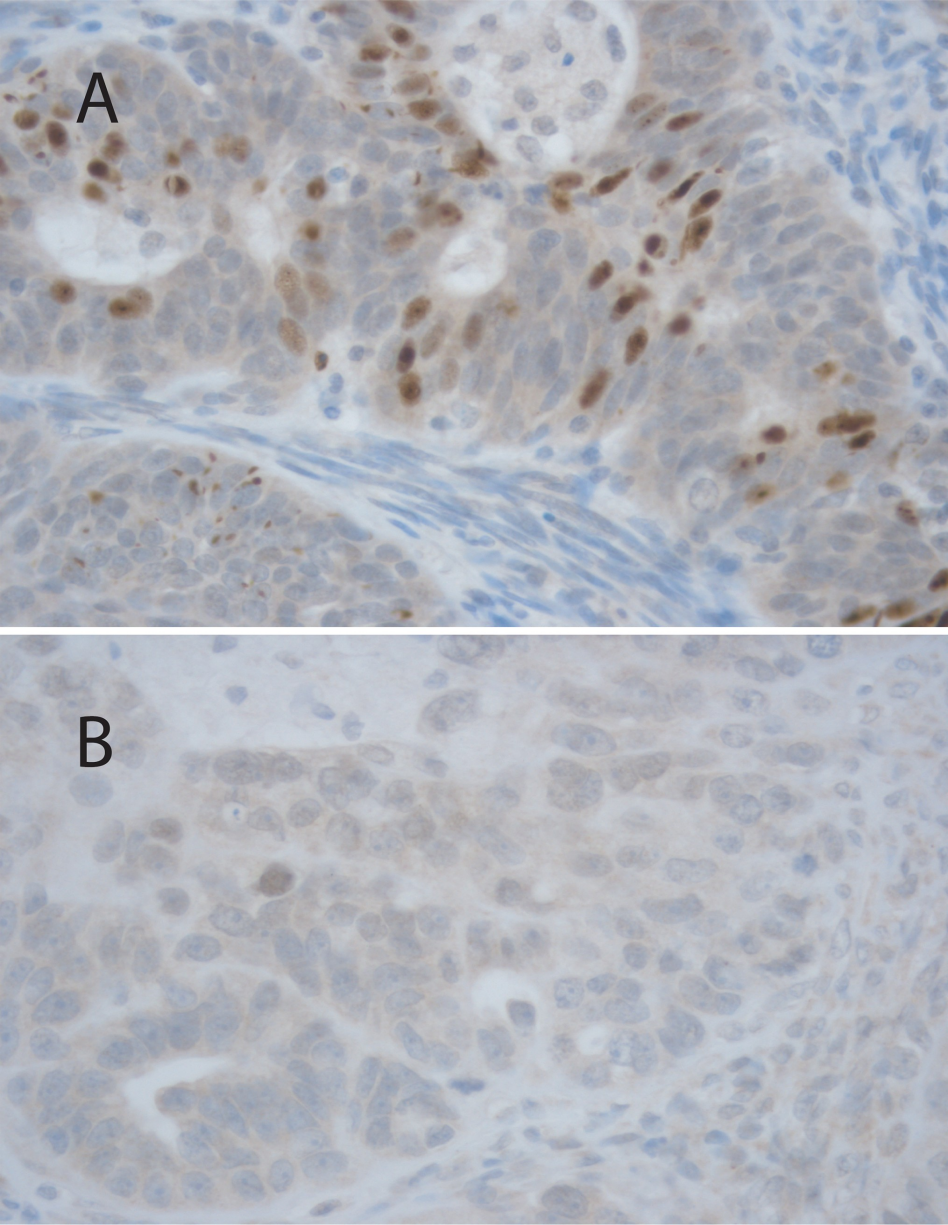
Images of immunohistochemical staining with high (A) and low (B) Hif-1α expression in ovarian cancer cells. Images are at 40 x magnification.

The tissue samples from tumors were embedded in paraffin and cut into 5-μm-thick sections. The sections were processed for hematoxylin-eosin and HIF-1α (Novus 1:75, United Kingdom) staining. An experienced pathologist selected the most representative samples, i.e. those with the most tumoral component in routine hematoxylin and eosin-stained slides, which were then examined with immunohistochemical staining (HIF-1α). The HIF-1α expression was analyzed from the nuclei of the epithelial OC cells (Fig 2) and was scored semiquantitatively using the immunoreactive score (IRS)[34,35]. In brief, IRS is the multiplication of staining intensity (0 = negative, 1 = weak, 2 = moderate and 3 = strong) and the description for percentage of positive cells (0 = negative, 1 = 1–10%, 2 = 11–50%, 3 = 51–75% and 4 = 76–100%). The median IRS value was used as a cutoff for low and high expression.

### Statistical analysis

SPSS for Windows (Version 22.0, 2013, SPSS Inc., Chicago, IL, USA) was used for statistical analysis. Values are presented as mean ± SD unless otherwise stated. The Intraclass correlation coefficient (ICC) was used in interobserver reproducibility analysis. For classified parameters, Kruskal–Wallis and Mann–Whitney U tests were used when appropriate. Chi-square test was used for frequency tables. *P* ≤ 0.05 was considered statistically significant. Analysis of receiver operating characteristic (ROC) curves was performed for DCE pharmacokinetic perfusion parameters to discriminate between low and high levels of HIF-1α expression. The area under the curve (AUC) was classified as “low” (0.5–0.7), “moderate to good” (0.7–0.9) and “very good to excellent” (0.9–1). The values of AUC, specificity, sensitivity, positive predictive value (PPV), negative predictive value (NPV) and cutoff value were calculated. The most appropriate cutoff values were determined according to the highest Youden Index (sensitivity + specificity -1) from estimate curves. Association of these cutoff values with HIF-1α expression was tested with Chi-square test.

## Results

A total of 38 patients were recruited. Three patients were excluded from DCE imaging analyses due to insufficient image quality: two due to movement artifacts and one because of a different scanning time. The five patients who received neoadjuvant chemotherapy were excluded from the histopathological analysis. Thus, altogether 30 patients with primary OC (mean age 66 years, range 47–86 years) were included (Table 1). Staging operation was performed within 7 ± 6 days after imaging. The largest diameter for the solid tumor component was 77 mm (range 23–233 mm). Intraclass correlation coefficient (ICC) were excellent for DCE perfusion parameters K^trans^, K_ep_ and V_e_ (K^trans^ L-ROI 0.994 and S-ROI 0.980; K_ep_ L-ROI 0.985 and S-ROI 0.921; V_e_ L-ROI 0.997 and S-ROI 0.964) but for V_p_ only good to moderate (L-ROI 0.621 and S-ROI 0.598).

### HIF-1α expression in ovarian cancer

In this cohort, HIF-1α expression was low in 26 (84%) and high in 5 (16%) patients (Fig 2). HIF-1α was low in 80% (4/5) of stage I, 100% (2/2) of stage II, 85.7% (12/14) of stage III and 80% (8/10) of stage IV tumors and correspondingly, in stages I–IV, high in 20% (1/5), 0% (0/2), 14.3% (2/14) and 10% (2/10). HIF-1α did not associate significantly with the FIGO stage (*P* = 0.900). In addition, HIF-1α expression was independent of patient’s age (*P* = 0.206) and CA-125 levels (*P* = 0.957) as well as of histological grade (*P* = 0.892) and subtype of the tumor (*P* = 0.952). Furthermore, the expression of HIF-1α did not associate with the result of surgical operation, response to treatment, platinum sensitivity or to tumor recurrence (Table 1).

### HIF-1α association with DCE parameters

**Fig 3:**
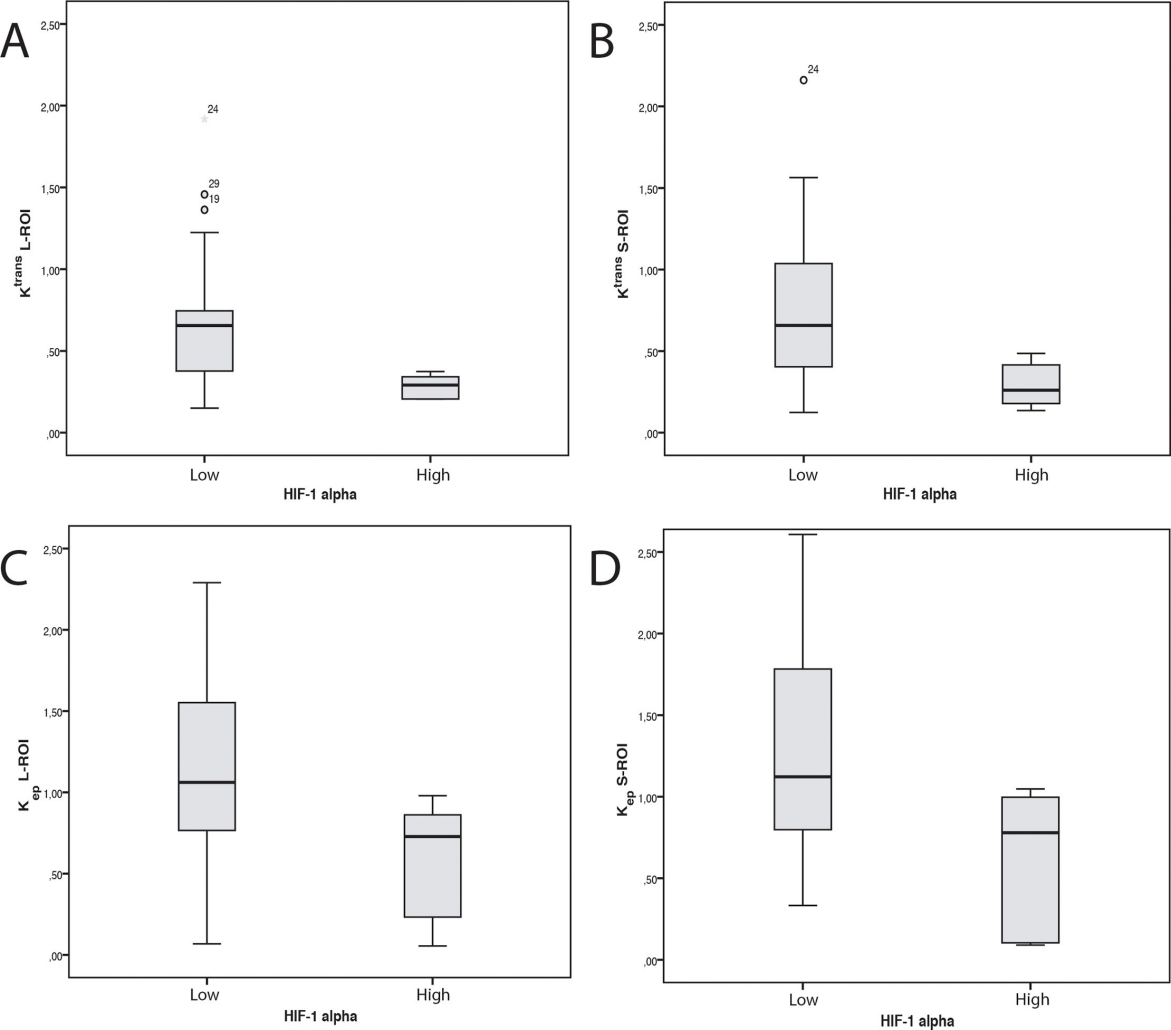
Imaging demonstrating the association between the expression of the hypoxia specific marker, hypoxia-inducible factor 1 alpha (HIF-1α) with K^trans^ and K_ep_ values. A) The association between HIF-1α expression with K^trans^ L-ROI (P = 0.021 B) with K^trans^ S-ROI (P = 0.018) C) with K_ep_ L-ROI (P = 0.032) and D) with K_ep_ S-ROI (P = 0.033). The HIF-1α expression dichotomized into low and high using the median immunoreactive score as the cutoff value.

**Fig 4:**
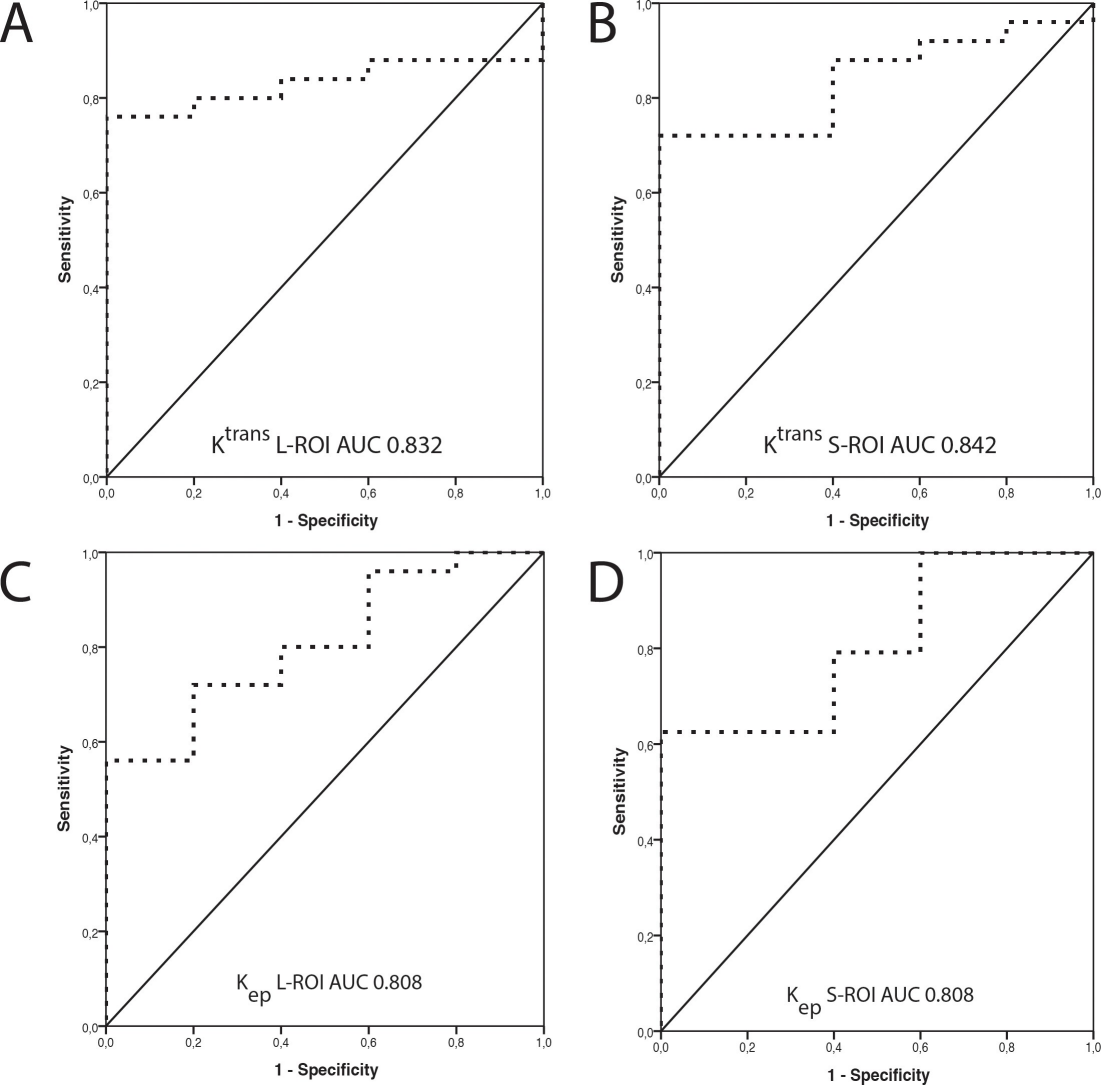
Analysis of ROC curves for the two DCE perfusion parameters, K^trans^ and K_ep_, in the discrimination between low and high HIF-1α expression. The analysis reveals the diagnostic accuracy of A) K^trans^ L-ROI (AUC = 0.832) B) K^trans^ S-ROI (AUC = 0.840), C) K_ep_ L-ROI (AUC = 0.808) and D) K_ep_ S-ROI (AUC = 0.808). HIF-1α expression dichotomized into low and high using the median immunoreactive score as the cutoff value.

HIF-1α expression was inversely associated with K^trans^ and K_ep_ values (Fig 3). Association was significant for both L- and S-ROIs (K^trans^ L-ROI *P* = 0.021and S-ROI *P* = 0.018; K_ep_ L-ROI *P* = 0.032 and S-ROI *P* = 0.033). HIF-1α expression did not associate with V_e_ or V_p_ values (Table 2). Analysis of the ROC curve demonstrated good accuracy for DCE parameters K^trans^ and K_ep_ in discriminating between low and high HIF-1α expression, with AUCs of 0.832 for K^trans^ L-ROI, 0.840 for K^trans^ S-ROI, 0.808 for K_ep_ L-ROI and 0.808 for K_ep_ S-ROI (Fig 4). A summary of AUC, cutoff values, sensitivity, specificity, PPV, NPV and Youden Index for K^trans^ and K_ep_ for distinguishing low and high HIF-1α is shown in Table 3. The following cutoff values were further used to dichotomize the K trans and K ep values into low or high: 0.375 (L-ROI), 0.487 (S-ROI) for Ktrans and 0.874 (L-ROI) and 1.071 (S-ROI) for Kep. These dichotomized DCE parameters proved to be statistically significantly associated with HIF-1α expression (K^trans^ L-ROI *P* = 0.003; K^trans^ S-ROI *P* = 0.003; K_ep_ L-ROI *P* = 0.047 and K_ep_ S-ROI *P* = 0.017).

**Table 2:** The associations between the expression of hypoxia-inducible factor 1alpha (HIF-1α) and various perfusion parameters.

	HIF-1α low	HIF-1α high	p-value
K^trans^ L-ROI	0.88 (0.96)	0.30 (0.07)	0.021
K^trans^ S-ROI	0.95 (1.12)	0.39 (0.14)	0.018
K_ep_ L-ROI	1.19 (0.58)	0.62 (0.46)	0.032
K_ep_ S-ROI	1.21 (0.54)	0.68 (0.43)	0.033
V_e_ L-ROI	85.07 (91.60)	50.74 (35.79)	0.388
V_e_ S-ROI	93.33 (102.46)	66.59 (48.22)	0.420
V_p_ L-ROI	9.43 (7.99)	15.30 (14.20)	0.419
V_p_ S-ROI	10.67 (8.87)	18.98 (15.50)	0.488

Results are mean ± standard deviation (SD). HIF-1α has been dichotomized using the median immunoreactive score as the cutoff value.

K^trans^ = the rate constant for transfer of contrast agent from plasma to extravascular, extracellular space (EES); K_ep_ = the rate constant for transfer of contrast agent from EES to plasma, V_e_ = contrast agent distribution volume, EES volume fraction, V_p_ = plasma volume fraction

**Table 3:** Analyses of receiver operating characteristic (ROC) curves of DCE perfusion parameters to discriminate between low and high expression of hypoxia-inducible factor 1 alpha (HIF-1α).

	AUC (95% CI)	Cut-off	Sensitivity (95% CI)	Specificity (95% CI)	PPV (95% CI)	NPV (95% CI)	Youden index
**K^trans^ L-ROI**	0.832 (0.691–0.973)	0.375	1.00 (0.57–1.00)	0.76 (0.57–0.89)	0.45 (0.21–0.72)	1.00 (0.83–1.00)	0.760
**K^trans^ S-ROI**	0.840 (0.693–0.987)	0.487	1.00 (0.57–1.00)	0.72 (0.52–0.86)	0.42 (0.19–0.68)	1.00 (0.82–1.00)	0.720
**K_ep_ L-ROI**	0.808 (0.629–0.987)	0.874	0.80 (0.38–0.96)	0.72 (0.52–0.86)	0.36 (0.15–0.64)	0.95 (0.75–0.99)	0.520
**K_ep_ S-ROI**	0.808 (0.624–0.993)	1.071	1.00 (0.57–1.00)	0.63 (0.43–0.79)	0.36 (0.16–0.61)	1.00 (0.80–1.00)	0.625

AUC = area under the curve; CI = confidence interval; PPV = positive predictive value, NPV = negative predictive value; Youden Index = sensitivity + specificity -1

## Discussion

This study investigated the association between the hypoxia specific marker, HIF-1α, and pharmacokinetic perfusion parameters measured by DCE MR imaging in OC. There was a significant inverse association between HIF-1α and K^trans^ and K_ep_ values. Based on the ROC analysis, K^trans^ and K_ep_ values proved to be good discriminators for identifying low and high HIF-1α expression in OC. In the patients examined in the present study, HIF-1α was, however, not associated with known clinical prognostic factors, such as stage, age, residual tumor in debulking surgery or primary treatment results.

K^trans^ is a rate constant reflecting the contrast agent’s flow from plasma to the interstitial space through vessel walls. K_ep_ is a rate constant of the opposite flow from EES to plasma. Certain features of blood vessel, such as maturity, vessel fenestrations and perivascular space, influence this movement and thus the values of these constants [36,37]. In solid malignant tumors, neoangiogenesis causes the cancer’s blood vessels to be immature, leaky and fragile. Consequently, malignant tumors have higher perfusion than their benign counterparts do.

Tumors with low oxygenation levels start to induce the expression of HIF-1α which is an important factor for tumor growth e.g. allowing the tumor cells to adapt to a hypoxic environment and regulating the production of pro-angiogenic factors [9]. Interestingly, in this study, K^trans^ and K_ep_ were inversely associated with HIF-1α expression. The published results between tumor oxygenation and DCE perfusion parameters in different types of malignancies are somewhat conflicting (Table 4). As far as we are aware, no prior studies have investigated this correlation in OC.

**Table 4:** Studies that have examined the relationships between oxygenation and DCE perfusion parameters.

	Tumor type	N	MRI	DCE variables	Histopathological variables	Hif-1α correlation
Borren[33]	prostate cancer	15	3 T	K^trans^, K_ep_	HIF-1α and HIF-2α	No correlation with K^trans^, K_ep_, MVD
Loncaster[25]	cervical cancer	35	1.5 T	Amplitude, K_ep_	Po_2_ histograph	Po_2_ inverse correlation with Kep
Halle[32]	cervical cancer	78	1.5 T	brix model, A_Brix_, K_ep_, K_el_	HIF-1α	Inverse correlation with A_Brix_
Berg[41]	endometrial cancer	164	1.5 T	blood flow, K^trans^, K_ep_, V_e_, Fb, IAUGC, ADC	HIF-1α	No correlation with Fb, K^trans^, K_ep_, Ve, IAUGC, ADC
Xie[29]	glioma	34	3 T	K^trans^, K_ep_, V_e_, V_p_	HIF-1α	Positive correlation with Ktrans and Ve
Awasthi[30]	glioma	76	1.5 T	rCBV, rCBF, K^trans^, K_ep_, V_e_	HIF-1α, vegf, prl-3, MMP-9	Positive with rCBV, and VEGF expression
Jensen[31]	glioblastoma	16	1.5 T, 3 T	F, E, PS, K^trans^, K_ep_, V_e_, V_b_, tc, α^–1^	HIF-1a, VEGF, CA-IX, GLUT-1	Positive with tc and V_b_
Present study	ovarian cancer	30	3 T	K^trans^, K_ep_, V_e_, V_p_	HIF-1α	Inverse association with K^trans^ and K_ep_

N = number of patients, MRI = magnetic resonance imaging, T = tesla, K^trans^ = a rate constant for transfer of contrast agent from plasma to extravascular, extracellular space (EES); K_ep_ = a rate constant for transfer of contrast agent from EES to plasma, A_Brix_ = amplitude, K_ep_ = the transfer rate of tracer from tissue to plasma, K_el_ = the clearance rate of the tracer from plasma, V_e_ = contrast agent distribution volume, EES volume fraction, Fb = blood flow, IAUGC = integrated area under the concentration time curve, V_p_ = plasma volume fraction, rCBV = relative cerebral blood volume, rCBF = relative cerebral blood flow, CER = contrast enhancement ratio, PR = pattern recognition technique, F = tumor blood flow, E = extraction fraction, PS = permeability surface area product, V_b_ = blood volume, tc = capillary transit time, and α^–1^ = capillary heterogeneity, HIF-1α = Hypoxia-inducible factor 1alpha, VEGF = vascular endothelial growth factor, CA-IX = carbonic anhydrase IX, and GLUT-1 = glucose transporter-1

In gliomas, Xie et al. showed that HIF-1α was correlated positively with K^trans^ and V_e_ values [29]. These investigators used stereotactic biopsies for histopathological samples, to minimize the mismatch of measurements. In another study with gliomas, a positive correlation was observed between HIF-1α and relative cerebral blood volume, but in that study, the expression level of HIF-1α did not correlate significantly with either K^trans^ or K^ep^ values [30]. Jensen et al. examined glioblastoma specimens and noted that HIF-1α correlated positively with blood volume and capillary transit time [31]. In their study, higher perfusion parameters were associated with worse outcome [31]. One explanation for the difference in the correlation direction between gliomas and OC might be the blood brain barrier. It is possible that the distribution of the contrast agent is different in OC as compared to brain tumors, in the latter case, the contrast has first to penetrate through the blood brain barrier.

On the other hand, studies in cervical cancer have shown that higher levels of tumor perfusion and permeability are associated with a better prognosis [38,39]. It has been postulated that highly enhancing tumors are better oxygenated [38,39]. Should that be the case, also the levels of HIF-1α should be lower when perfusion parameters are high as HIF-1α starts to increase in a hypoxic environment. This proposal would help to interpret the results of this study where an inverse association was detected between HIF-1α and DCE perfusion parameters.

In part paralleling the results of the present study, Loncaster et al. examined a cohort of 35 patients with cervical cancer and reported that oxygenation status correlated inversely with K_ep_ levels [25]. The authors used the Eppendorf Po_2_ histograph method for evaluating the oxygenation level and the possible correlation between oxygenation and K^trans^ levels was not studied. Another study in cervical cancer showed that HIF-1α upregulation was associated with low Amplitude in the Brix model (A_Brix)_ [32]_._ Even when using a different model for interpreting the pharmacokinetic data, they also obtained an inverse association between HIF-1α and A_Brix_, a parameter reflecting interstitial and blood volume [32,40].

In a study of prostate cancer patients, neither HIF-1α nor -2α correlated with K^trans^ or K_ep_ values [33], but there were only 15 patients in the final analysis. When patients with endometrial cancer were examined, the expression levels of HIF-1α did not show any correlation with K^trans^ or K_ep_ values, however a low tumor blood flow was associated with a hypoxia gene signature and HIF-1α expression [41].

It has been shown that cancer can elevate HIF-1α levels in both an oxygen-dependent and independent manner. HIF-1α expression increases through the oxygen-dependent pathway in hypoxic situations; it facilitates adaptation to oxygen deprivation, for example by regulating the expression of several genes which control glucose uptake, metabolism, angiogenesis, erythropoiesis, cell proliferation, and apoptosis [9,10]. In the hypoxia-independent pathway, increased oncogenic signaling in cancer cells induces HIF-1α expression [42–44]. These two pathways may partly explain the discrepancy in the literature regarding the association between HIF-1α expression and K^trans^ or K_ep_ in different tumors. Hypothetically, HIF-1α expression may be more dependent on the oxygen-dependent pathway in fast-growing, highly proliferative, tumors such as OC than in slow-growing tumors.

In glioma studies, the expression of HIF-1α has been correlated positively with tumor grade [29,30], which is a strong prognostic factor for survival in gliomas. In the present study of patients with OC, we observed no correlation of HIF-1α expression with either clinical prognostic factors or grade. This is in accordance with some other studies in OC [11,45]. Furthermore, in the present study, we did not detect any correlation between primary treatment results and HIF-1α expression, although this may be related to the small size of our cohort.

The results of the present study suggest that K^trans^ and K_ep_ are good indicators for differentiating tumors with low vs. high HIF-1α expression levels in OC. In the ROC curve analysis, K^trans^ and K_ep_ displayed good accuracy to detect high HIF-1α expression. PPV was slightly better for K^trans^ than for K_ep_ values. When we further dichotomized the DCE parameters into low or high they proved to be significantly associated with the HIF-1α expression. No earlier studies have calculate cutoff points for DCE parameters to determine high HIF-1α expression in OC.

There are some limitations to these findings. Firstly, the sample size was only 30 patients. Secondly, there is a possibility of some mismatch between the site of histopathological samples and ROI placements. In OC, the tumors are located deep in the pelvis, preventing any kind of stereotactic biopsy for sampling the cancer. Histopathological samples were obtained from radically resected tumors, from which the pathologist chose the most representative part of the tumor for immunohistochemical analyses. S-ROI has been delineated in the same area of tumor as the pathological sample, but still the possibility of mismatch remains. In our study protocol, the acquisition time was 6.7sec/stack in the perfusion scan for 51 slices with an acquisition matrix of 267*387. T1 maps were not included because the imaging protocol was already time-consuming. The AIF shape was inspected individually as being accurate for all patients. B1 correction was not performed. In future studies, a shorter temporal resolution could yield data that are more robust. In addition, when comparing results from different investigators and institutions, only results from studies that have used similar MR scanners, scanning protocols and DCE analysis are directly comparable.

In conclusion, the results of the present study suggest that in patients with OC, the expression level of hypoxia-inducible factor 1 alpha (HIF-1α) is inversely associated with tumor pharmacokinetic DCE-MRI perfusion parameters K^trans^ and K_ep_. If it were possible to detect the poorly oxygenated tumors preoperatively, this could help the physician to make decisions of treatment modalities and encourage the efficient use of hypoxia-modifying therapies [43,46]. Although DCE MRI may become a valuable imaging tool for detailed OC tumor characteristic screening, more trials will need to be completed before it becomes part of the clinical routine.

## Supporting information

S1 Table Characteristics and measurements of the study population. (XLSX)
